# Community-Engaged Language and Cultural Adaptation of a Mobile Health Behavioral Incontinence Management Program

**DOI:** 10.1093/geroni/igaf122.4369

**Published:** 2025-12-31

**Authors:** Alison Huang, Celia Kaplan, Alayne Markland, Claudia Vila Manes, Jenny Chen, Joyce Cheng, Joana Mattero, Leah Karliner

**Affiliations:** University of California, San Francisco, San Francisco, California, United States; University of California, San Francisco, San Francisco, California, United States; University of Utah School of Medicine, Salt Lake City, Utah, United States; University of California, San Francisco, San Francisco, California, United States; University of California, San Francisco, San Francisco, California, United States; Chinese Community Health Resource Center, San Francisco, California, United States; Mission Neighborhood Centers, San Francisco, California, United States; University of California, San Francisco, San Francisco, California, United States

## Abstract

Language and cultural barriers prevent many midlife and older ethnic minority women from accessing first-line behavioral management interventions for urinary incontinence, despite these techniques’ proven effectiveness. This interdisciplinary, community-engaged project adapted an interactive smartphone program for behavioral incontinence management (MyHealtheBladder) to enable its use by midlife and older Latina and Chinese American women with limited English proficiency. Using Barrera and Castro’s cultural adaptation framework, we engaged geriatrician and incontinence experts, health communication scientists, and community advocates, in addition to Latina and Chinese American women with incontinence across the aging spectrum. The adaptation process involved systematic review and translation of program content, iterative discussion and refinement by community partners, and content pilot testing in Spanish- and Chinese-speaking women aged 46-77 years with weekly incontinence. Community engagement revealed universal challenges in discussing clinical and anatomical concepts related to incontinence, while identifying culture-specific considerations including varying comfort levels with bladder/bowel discussions, preferences for medical versus colloquial terminology, and differing expectations regarding patient self-management versus clinician consultation. The adaptation process resulted in multiple changes to the language or terminology used to describe toileting activities, pelvic anatomy, and patient-clinician interactions, as well as patient case examples used to reinforce behavioral management techniques. Results demonstrate how meaningful community partnership can identify both shared and distinct cultural needs in health intervention adaptation. The project’s community-engaged approach addressed language barriers while honoring cultural values and communication preferences, offering a model for adapting digital health interventions for diverse aging populations facing similar barriers for sensitive health conditions.

